# A pragmatic randomised controlled trial referring to a Personalised Self-management SUPport Programme (P-SUP) for persons enrolled in a disease management programme for type 2 diabetes mellitus and/or for coronary heart disease

**DOI:** 10.1186/s13063-021-05636-4

**Published:** 2021-09-27

**Authors:** Uwe Konerding, Marcus Redaèlli, Karolin Ackermann, Sibel Altin, Sebastian Appelbaum, Bianca Biallas, August-Wilhelm Bödecker, Suzan Botzenhardt, Chloé Chermette, Martin Cichocki, Iris Dapper, Katja Dehnen, Christian Funke, Angeli Gawlik, Lisa Giesen, Johannes Goetz, Christian Graf, Bernd Hagen, Martina Heßbrügge, Phillip Hendrick Höhne, Jens Kleinert, Helene Könnecke, Lucas Küppers, Nicole Kuth, Lion Lehmann, Claas Lendt, Khalid Majjouti, Yeliz Nacak, Aliza Neuhausen, Larisa Pilic, Lara Schneider, Maximilian Scholl, Dusan Simic, Andreas Sönnichsen, Anika Thielmann, Ines Van der Arend, Frank Vitinius, Birgitta Weltermann, Dorothea Wild, Stefan Wilm, Stephanie Stock

**Affiliations:** 1 Trimberg Research Academy, University of Bamberg, D-96045 Bamberg, Germany; 2 Department of Psychology and Psychotherapy, Witten/Herdecke University, Alfred-Herrhausen-Straße 50, D-58448 Witten, Germany; 3 Institute of Health Economics and Clinical Epidemiology, University Hospital Cologne (Institut für Gesundheitsökonomie und Klinische Epidemiologie, Universitätsklinikum Köln), D-50924 Köln, Germany; 4 Department of Psychosomatics and Psychotherapy, University Hospital Cologne (Klinik und Poliklinik für Psychosomatik und Psychotherapie, Universitätsklinikum Köln), Weyertal 76, 50931 Köln, Germany; 5 General Local Health Insurance, Rheinland/Hamburg (Allgemeine Ortskrankenkasse, Rheinland/Hamburg), Kasernenstraße 61, D-40213 Düsseldorf, Germany; 6 Institute of Movement Therapy and Movement-oriented Prevention and Rehabilitation, German Sport University Cologne (Institut für Bewegungstherapie und bewegungsorientierte Prävention und Rehabilitation, Deutsche Sporthochschule Köln), Am Sportpark Müngersdorf 6, D-50933 Köln, Germany; 7 Teaching Unit of General Practice, University Hospital Cologne (Schwerpunkt Allgemeinmedizin, Universitätsklinikum Köln), D-50924 Köln, Germany; 8 Institute of General Practice, University Duisburg-Essen (Institut für Allgemeinmedizin, Universität Duisburg-Essen), Hufelandstr. 55, D-45122 Essen, Germany; 9 Institute of Psychology, German Sport University Cologne (Institut für Psychologie, Deutsche Sporthochschule Köln), Am Sportpark Müngersdorf 6, D-50933 Köln, Germany; 10 Department of General Practice and Family Medicine, Medical University of Vienna (Abteilung für Allgemeinmedizin und Familienmedizin, Medizinischen Universität Wien), Kinderspitalgasse 15/1.Stock, A-1090 Wien, Austria; 11 Institute of General Practice, Heinrich Heine University Düsseldorf (Institut für Allgemeinmedizin, Heinrich-Heine-Universität Düsseldorf), Post Office Box 10 10 07, D-40001 Düsseldorf, Germany; 12 Barmer Health Insurance (Barmer Krankenversicherung), BARMER, Heerdter Lohweg 35, D-40549 Düsseldorf, Germany; 13 Central Research Institute of Ambulatory Health Care in Germany (Zentralinstitut für die Kassenärztliche Versorgung in Deutschland), Salzufer 8, D-10587 Berlin, Germany; 14 Institute of Family Medicine and General Practice, University of Bonn (Institut für Hausarztmedizin, Universität Bonn), Venusberg-Campus 1, D-53127 Bonn, Germany; 15 Teaching Area of General Practice, University Hospital RWTH Aachen (Lehrgebiet für Allgemeinmedizin Uniklinik RWTH Aachen), Pauwelsstraße 30, D-52074 Aachen, Germany

**Keywords:** Health services research, Type 2 diabetes mellitus, Coronary heart disease, Peer support group, Self-management, Study protocol, Pragmatic RCT, Pragmatic randomised controlled trial

## Abstract

**Background:**

Type 2 diabetes mellitus (T2DM) and coronary heart disease (CHD) are two chronic diseases that cause a tremendous burden. To reduce this burden, several programmes for optimising the care for these diseases have been developed. In Germany, so-called disease management programmes (DMPs), which combine components of Disease Management and the Chronic Care Model, are applied. These DMPs have proven effective. Nevertheless, there are opportunities for improvement. Current DMPs rarely address self-management of the disease, make no use of peer support, and provide no special assistance for persons with low health literacy and/or low patient activation. The study protocol presented here is for the evaluation of a programme that addresses these possible shortcomings and can be combined with current German DMPs for T2DM and CHD. This programme consists of four components:

1. Meetings of peer support groups
2. Personalised telephone-based health coaching for patients with low literacy and/or low patient activation
3. Personalised patient feedback
4. A browser-based web portal

**Methods:**

Study participants will be adults enrolled in a DMP for T2DM and/or CHD and living in North Rhine-Westphalia, a state of the Federal Republic of Germany. Study participants will be recruited with the assistance of their general practitioners by the end of June 2021. Evaluation will be performed as a pragmatic randomised controlled trial with one intervention group and one waiting control group. The intervention group will receive the intervention for 18 months. During this time, the waiting control group will continue with usual care and the usual measures of their DMPs. After 18 months, the waiting control group will also receive a shortened intervention. The primary outcome is number of hospital days. In addition, the effects on self-reported health-state, physical activity, nutrition, and eight different psychological variables will be investigated. Differences between values at month 18 and at the beginning will be compared to judge the effectiveness of the intervention.

**Discussion:**

If the intervention proves effective, it may be included into the DMPs for T2DM and CHD.

**Trial registration:**

The study was registered in the German Clinical Trials Registry (Deutsches Register Klinischer Studien (DRKS)) in early 2019 under the number 00020592. This registry has been affiliated with the WHO Clinical Trials Network (https://www.drks.de/drks_web/setLocale_EN.do) since 2008. It is based on the WHO template, but contains some additional categories for which information has to be given (https://www.drks.de/drks_web/navigate.do?navigationId=entryfields&messageDE=Beschreibung%20der%20Eingabefelder&messageEN=Description%20of%20entry%20fields ). A release and subsequent number assignment only take place when information for all categories has been given.

**Supplementary Information:**

The online version contains supplementary material available at 10.1186/s13063-021-05636-4.

## Administrative information

**Table:** 

Title {1}	A Pragmatic Randomised Controlled Trial referring to a Personalised Self-management SUPport Programme (P-SUP) for persons enrolled in a disease management programme for type 2 diabetes mellitus and/or for coronary heart disease
Trial registration {2a and 2b}.	The study was registered in the DRKS at the beginning of 2019 under the number 00020592. Since October 2008 the DRKS has been maintained as a primary registry of the World Health Organization (WHO) and thus fulfils the requirements of the International Committee of Medical Journal Editors.
Protocol version {3}	P-SUP_Study Protocol_Version _03
Funding {4}	The development and evaluation of the intervention are fully funded by the Federal Joint Committee (Gemeinsamer Bundesausschuss (G-BA)). https://innovationsfonds.g-ba.de/projekte/neue-versorgungsformen/p-sup-personalisiertes-selbstmanagement-unterstuetzungsprogramm.285
Author details {5a}	Konerding, Uwe (1,2); Redaèlli, Marcus (3); Ackermann, Karolin (4); Altin, Sibel (5); Appelbaum, Sebastian (1,2); Biallas, Bianca (6); Bödecker, August-Wilhelm (7); Botzenhardt, Suzan (8); Chermette, Chloé (9); Cichocki, Martin (10); Dapper, Iris (7); Dehnen, Katja (8); Funke, Christian (11); Gawlik, Angeli (9); Giesen, Lisa (3); Goetz, Johannes (3); Graf, Christian (12); Hagen, Bernd (13); Heßbrügge, Martina (8); Höhne, Phillip Hendrick (5); Kleinert, Jens (9); Könnecke, Helene (3); Küppers, Lucas (14); Kuth, Nicole (15); Lehmann, Lion (7); Lendt, Claas (6); Majjouti, Khalid (14); Nacak, Yeliz (4); Neuhausen, Aliza (4); Pilic, Larisa (7); Schneider, Lara (4); Scholl, Maximilian (6); Simic, Dusan (3); Sönnichsen, Andreas (10); Thielmann, Anika (14); Van der Arend, Ines (15); Vitinius, Frank (4); Weltermann, Birgitta (14); Wild, Dorothea (14); Wilm, Stefan (11); Stock, Stephanie (3) 1) Trimberg Research Academy (TRAc Bamberg), University of Bamberg 2) Department of Psychology and Psychotherapy, Witten/Herdecke University 3) Institute of Health Economics and Clinical Epidemiology, (Institut für Gesundheitsökonomie und Klinische Epidemiologie (IGKE Cologne)) University Hospital Cologne 4) Department of Psychosomatics and Psychotherapy, University Hospital Cologne, (Klinik und Poliklinik für Psychosomatik und Psychotherapie, Universitätsklinikum Köln (KPPP Cologne)) 5) General Local Health Insurance, Rheinland/Hamburg (Allgemeine Ortskrankenkasse, Rheinland/Hamburg (AOK RH)) 6) Institute of Movement Therapy and Movement-oriented Prevention and Rehabilitation, German Sport University Cologne (Institut für Bewegungstherapie und bewegungsorientierte Prävention und Rehabilitation, Deutsche Sporthochschule Köln (DSHS Movement)) 7) Teaching Unit of General Practice, University Hospital Cologne (Schwerpunkt Allgemeinmedizin Universitätsklinikum Köln (SAM Cologne) 8) Institute of General Practice, University Duisburg-Essen (Institut für Allgemeinmedizin, Universität Duisburg-Essen (IFAM Essen)) 9) Institute of Psychology, German Sport University Cologne (Institut für Psychologie, Deutsche Sporthochschule (DSHS Psychology)) 10) Department of General Practice and Family Medicine, Medical University Vienna (Abteilung für Allgemeinmedizin und Familienmedizin, Medizinischen Universität Wien (AAF Vienna)) 11) Institute of General Practice, Heinrich Heine University Düsseldorf (Institut für Allgemeinmedizin, Heinrich-Heine-Universität Düsseldorf (ifam Düsseldorf)) 12) Barmer Health Insurance (Barmer Krankenversicherung (BARMER)) 13) Central Research Institute of Ambulatory Health Care in Germany (Zentralinstitut für die Kassenärztliche Versorgung in Deutschland (Zi)) 14) Institute of Family Medicine and General Practice, University of Bonn (Institut für Hausarztmedizin, Universität Bonn (IFAM Bonn)) 15) Teaching Area of General Practice, University Hospital RWTH Aachen (Lehrgebiet für Allgemeinmedizin Uniklinik RWTH Aachen (AMED Aachen))
Name and contact information for the trial sponsor {5b}	The trial sponsor is the G-BA, the highest decision-making committee of the joint self-government of physicians, dentists, hospitals and health insurance funds in Germany. The postal address is: Gemeinsamer Bundesausschuss (G-BA) Gutenbergstraße 13 10587 Berlin
Role of sponsor {5c}	The trial sponsor plays no role in study design; collection, management, analysis and interpretation of data; writing of the report; or the decision to submit the report for publication. The trial sponsor also has no authority over any of these activities.

## Introduction

### Background and rationale {6a}

#### Background

Type 2 diabetes mellitus (T2DM) and coronary heart disease (CHD) are both chronic diseases with a high impact on public health. Analyses based on data of the European Health Interview Survey show that, in 2014, 7.7% of the adult German population suffered from diabetes mellitus, in most cases T2DM [1], and 4.8% from CHD [2]. The worldwide prevalence of diabetes mellitus in adult persons was estimated to be 8.5% for 2014 [3]. In comparison, for 1980, this prevalence was only 4.7% [3]. This indicates an enormous increase of diabetes mellitus within the last decades. This dramatic rise is largely due to the rise in T2DM [3]. There are no valid estimates of the worldwide prevalence of CHD. However, cardiovascular diseases, which include CHD, are considered to have been the cause of 17.9 million worldwide deaths in 2016, which accounted for 31% of all worldwide deaths in that year [4]. Together, T2DM and cardiovascular disease including CHD are major causes of premature death, disability, productivity loss, and decrease in quality of life [5]. Accordingly, T2DM and CHD contribute substantially to the medical, social, and economic burden caused by non-communicable diseases, and this burden constitutes a major challenge for healthcare systems around the world [6].

The burden caused by diseases like T2DM and CHD has motivated the development of approaches for effective coping with chronic diseases. The two best-known approaches are Disease Management and the Chronic Care Model [7]. Disease Management aims to provide disease-specific, evidence-based, coordinated, and integrated care for chronic sufferers from a defined condition with the goal of improving outcomes and life expectancy and of containing costs through pro-active care. An integral part of Disease Management is patient education and the activation of patients to perform self-management. There is international evidence for the effectiveness of Disease Management for both T2DM and CHD [8]. The Chronic Care Model emerged through summarisation of the results of a review of previous effective approaches for chronic disease care [9]. This model recommends the combination of several components of health care with each other. These components are ‘effective team care and planned interactions; self-management support bolstered by more effective use of community resources; integrated decision support; and patient registries and other supportive information technology’ [10]. As in the case of Disease Management, the Chronic Care Model has also proven to be effective for T2DM and CHD [10].

Disease Management and the Chronic Care Model constitute the conceptual basis of Germany’s Disease Management Programmes (DMPs). In these programmes, recommendations provided by Disease Management for the interaction between physicians and patients are combined with recommendations provided by the Chronic Care Model for the design of those parts of the health system that are relevant for the treatment of T2DM and CHD. Special hallmarks of the German DMPs are that they are patient-centred and physician-based, and that they include all patients with a certain condition in a preventive approach rather than targeting a high-risk population. There is evidence that the German DMPs improve clinical outcomes, quality of life and survival [11, 12]. However, current DMPs contain almost no components that support self-management and, thereby, health-promoting lifestyle behaviour. The only structured support of self-management consists in patient education classes [13]. Neglecting self-management in this way could be fatal because certain kinds of lifestyle behaviour, especially healthy nutrition and regular physical activity, have very favourable effects in cases of T2DM and CHD. They improve physical fitness, glycaemic control, blood pressure control and quality of life in patients [14]. For this reason, the effectivity of DMPs could increase substantially if they are extended to include components for enhancing self-management.

One approach for enhancing self-management is peer support, i.e. support from persons suffering from the same disease. This kind of support has several advantages compared to support provided by professionals. It is non-hierarchical, combines the benefits of both receiving and giving social support, provides prolonged emotional support, and affords opportunity to share similar life experiences. There is, in fact, extensive empirical evidence for the effectiveness of peer support for various health conditions, across age groups, social status and settings [15–17]. In addition to this, peer support has a further important advantage: it costs less than support provided by professionals. DMPs could therefore benefit from the integration of peer support components.

Other factors that influence self-management are health literacy and patient activation. Health literacy is the ability to seek, access, understand and apply information regarding one’s own health condition [18]. Patient activation is a multidimensional construct consisting of the dimensions ‘belief’, ‘knowledge’ and ‘skills’. Belief refers to the patients’ belief that they have an important role to play in shaping the treatment of their medical conditions, knowledge stands for the patients’ knowledge of how to manage their medical conditions and skills refers to the patients’ skills and behavioural repertoire in terms of acting according to their belief and knowledge [19]. There is extensive empirical evidence that health-related behaviour and medical outcomes are positively influenced by health literacy [20, 21] and patient activation [22]. These findings are especially relevant for the design of health care in Germany, as empirical evidence indicates that more than 50% of the German population have low health literacy [23]. Altogether, these findings suggest that the effectivity of DMPs in Germany could increase if components that specifically address the needs of patients with low health literacy or low patient activation are added.

As physical activity and nutrition are essential components of care for T2DM and CHD, findings regarding interventions designed to affect these two kinds of behaviour in persons with these diseases are also relevant. Hardly any research regarding the effectiveness of interventions for changing nutritional behaviour exists. However, such research does exist for physical activity and this research mainly refers to T2DM. There are several evaluation studies regarding interventions for promoting physical activity in persons with T2DM and even several reviews of these evaluation studies. The authors of a recent review of reviews of such evaluation studies identified 18 reviews referring to 113 separate trials [24]. From the results of these reviews, the authors inferred that two intervention components in particular help to promote physical activity: (1) giving feedback and (2) helping to integrate physical activity into daily life.

#### Rationale

The intervention presented here aims at removing the insufficiencies elaborated above and at applying the lessons that can be learned from previous research. The complete intervention will consist of four components:

1. Virtual and/or real meetings of peer support groups (PSGs)
2. Personalised telephone-based health coaching for patients with low literacy and/or low patient activation
3. Personalised patient feedback
4. A browser-based web portal

The PSGs will be groups of persons suffering from T2DM and/or CHD. These groups will be organised by persons also suffering from at least one of these diseases. The groups will meet weekly either virtually or in person. One major purpose of these groups will be to provide peer support with regard to self-management. To some extent, this support will consist in mutual exchange of advice on how health-promoting behaviour can be integrated into daily life. Personalised telephone-based health coaching will only be provided to participants with low health-literacy and/or low patient activation as assessed by means of a questionnaire. The purpose of this coaching will be to compensate for deficits that people with these characteristics have with regard to their self-management. Specifically, the telephone-based health coaching will aim at improving patients’ motivational and volitional control regarding integration of health-promoting forms of behaviour into their daily lives. The personalised patient feedback will consist in information about the patient’s current medical condition and is intended to aid individual adjustment of self-management. The browser-based web portal will offer information designed to help with self-management. In addition, this portal will contain modules designed to increase participants’ motivation regarding integration of health-promoting forms of behaviour into their daily lives.

## Objectives {7}

### Primary hypothesis

P-SUP reduces the number of hospital days.

### Secondary hypotheses

**Fig. 1:**
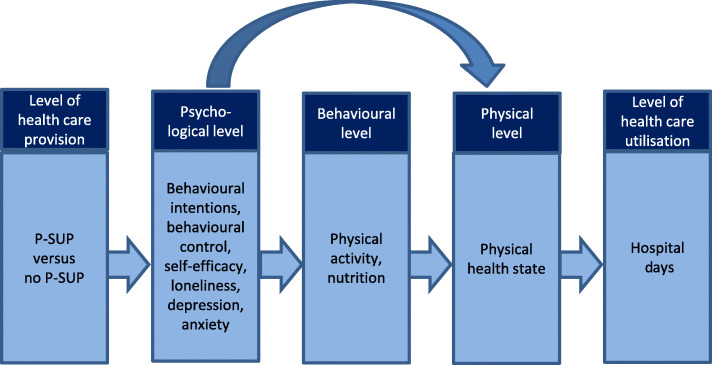
Theoretical frame

The secondary hypotheses are based on a theoretical frame that outlines how the effect of the intervention on number of hospital days is mediated. According to this frame, the mediating variables can be assigned to three different levels: (1) the physical level, which refers to the physical health state experienced by the study participants; (2) the behavioural level, which refers to the health-related behaviour performed by the study participants; and (3) the psychological level, which refers to the variables that reflect the way the study participants think and feel. If one adds the realm of health care provision, in which the intervention takes place, and the realm of health care utilisation, which includes hospital days, a five-level frame results (see Fig. 1). According to this theoretical frame, two causal pathways exist along which the intervention will affect the number of hospital days. The first pathway is a chain of causal effects from level to level. On the second pathway, the behavioural level is left out and replaced by a direct link from the psychological level to the physical level (see Fig. 1).

In addition to the number of hospital days, the intervention will also be evaluated with regard to variables that are assumed to mediate the effect of the intervention on the number of hospital days. These variables are self-reported physical health, which is assigned to the physical level; self-reported physical activity and self-reported nutrition, which are both assigned to the behavioural level; and eight variables belonging to the psychological level. The eight psychological variables are:

1. Intention to perform physical activity to the extent recommended by the World Health Organisation [26]
2. Perceived behavioural control with regard to physical activity of this extent
3. Intention to perform at least five of the nine items of health-promoting nutritional behaviour described in the questionnaire
4. Perceived behavioural control with regard to performing health-promoting nutritional behaviour of this extent
5. Self-efficacy with regard to health-related self-management,
6. Loneliness
7. Depression
8. Anxiety

Five of the eight psychological variables (i.e. intention with regard to physical activity, perceived behavioural control with regard to physical activity, intention with regard to nutritional behaviour, perceived behavioural control with regard to nutritional behaviour and self-efficacy with regard to health-related self-management) are assumed to affect behaviour and, mediated by this, experienced physical health. The remaining three psychological variables (i.e. loneliness, depression and anxiety) are assumed to affect experienced physical health directly. It is assumed that all the variables just mentioned (i.e. experienced physical health, the two behavioural variables and the eight psychological variables) will be positively affected by the intervention. Correspondingly, it is assumed that 18 months after the start of P-SUP, persons participating in this intervention will score better values on these variables than persons not participating in the intervention.

## Trial design {8}

The trial will be a pragmatic randomised controlled trial with one intervention and one waiting control group. Participants of the control group will receive the P-SUP-programme later than participants of the intervention group. The trial is pragmatic in that it will be performed in the exact setting in which it is ultimately intended to be applied, and in that all resources used will be available if the intervention is ultimately integrated into usual care. The allocation ratio of intervention to control is 6:7 because the drop-out rate for the intervention group is expected to be 30% and, for the control group, 40%. The trial is a superiority study [27] in which the superiority of the intervention to the comparator is to be demonstrated.

## Methods: participants, interventions and outcomes

### Study setting {9}

The study will be conducted jointly by several collaborating institutions in Germany. These institutions are IGKE Cologne, AMED Aachen, IFAM Bonn, SAM Cologne, ifam Düsseldorf, IFAM Essen, KPPP Cologne, DSHS Psychology and DSHS Movement. IGKE Cologne, AMED Aachen, IFAM Bonn, SAM Cologne, ifam Düsseldorf and IFAM Essen will approach general practices and recruit general practitioners (GPs), who will, in turn, recruit eligible patients. A substantial part of the intervention will be performed digitally or via telephone. The remaining part will consist in meetings of PSGs. The first PSG meetings will be held virtually via video conference tools. If the COVID-19 pandemic allows, later meetings can be held in reality. These meetings will be held either outdoor or in rooms that will be specifically rented for the meetings and will usually not belong to institutions providing any kind of health care. The meetings will be organised by selected patients, i.e. the PSG leaders. In some of the meetings, sports therapists or other experts will support the PSG leaders.

### Eligibility criteria {10}

All general practices in the study region will be eligible as long as they are willing to collaborate. Inclusion criteria for patients will be (1) being a patient of one of the included general practices, (2) being 18 years of age or older, (3) being enrolled in a DMP for T2DM and/or for CHD and (4) being a resident of North Rhine-Westphalia. Exclusion criteria will be (1) insufficient mastery of the German language, (2) severe cognitive or physical impairments and (3) severe comorbidities. The PSG leaders will be selected from the patients by the collaborating GPs. Selection criteria for PSG leaders will be aptitude for the role of a PSG leader as assessed by the GP and the patient’s willingness to assume this role. Patients selected as a PSG leader will undergo a special training course comprising four meetings each lasting half a day. The telephone coaches will not be peers. They will be employees of the KPPP Cologne who have been trained for the study in theoretical basics such as the underlying theory of telephone-based health coaching and the technique of Motivational Interviewing. Moreover, the telephone coaches have also received practical training in conducting telephone calls via exercises with actors.

### Who will take informed consent? {26a}

The GPs who recruit the patients will inform them about the project using informational material provided by the IGKE Cologne and will obtain their informed consent. The informational material contains information about the objective of the project, the contents of the project, the inclusion and exclusion criteria, the project’s benefits and risks for the participants, data processing, data security and the participants’ rights, especially the right of withdrawing from the project at any time. Moreover, the informational material also contains addresses of the persons responsible for the project and of the agencies who are responsible for monitoring data security (see Additional File 1).

### Additional consent provisions for collection and use of participant data and biological specimens {26b}

Not applicable.

### Interventions

#### Explanation for the choice of comparators {6b}

The participants in the control group will receive their usual care together with the DMPs in which they are enrolled. This regimen will be chosen as the comparator because P-SUP is intended as a supplement to DMPs.

#### Intervention description {11a}

As mentioned above (Background and rationale {6a} section), the intervention will consist of four components:

1) Virtual and/or real meetings of PSGs

2) Personalised telephone-based health coaching for patients with low literacy and/or low patient activation

3) Personalised patient feedback

4) A browser-based web portal.

The PSGs will be organised by persons who also suffer from T2DM and/or CHD. These persons (i.e. the PSG leaders) will be trained for their task before the intervention begins for all participants. The contents of this training have been developed by experts together with patients, subjected to a pre-test with members of the target group and revised based on the results of this pre-test. The training itself will be given by specialised professionals. To maintain low threshold, these will be medical assistants, senior medical students or sports therapists who have been trained by experts. The training will be given in four meetings lasting half a day each. At the start of the training, the general objective of the intervention and the role of the PSG leaders will be elucidated. The remainder of the course will consist of two sections: one section focusing on living with chronic illness; the other on group dynamics and motivation. Among other things, methods for coping with stress, especially psychological stress in chronic illnesses, and the influence of health on personality will be discussed in the section on living with chronic illness. The aim of the section on group dynamics and motivation will be to support the PSG leaders as facilitators, mediators and motivators for the group and its individual participants. In addition, the PSG leaders will receive instructions on how emergencies can be recognised and handled. All topics will be introduced in short lectures and practised interactively in simulations. The PSG leaders will also receive a manual for follow-up and consolidation.

The PSGs will consist of 4 to 6 participants. These will be selected so that their places of residences are as close together as possible. The groups will meet weekly for the full duration of the intervention. As a rule, each meeting will last 90 min. However, the PSGs will be allowed to change the length of the meetings as they see fit. The schedule of the meetings can also be set by the PSGs. As soon as the development of the pandemic allows, the PSGs can choose between face-to-face and digital meetings. The meetings will focus on physical exercise. Individual physical exercises tailored to the abilities / possibilities of the participants will be demonstrated. The selected exercises will address coordination skills, endurance and strength development. Every second month, an external expert will visit the PSG and give a talk on a topic of relevance for people with T2DM and/or CHD. The topics will cover nutrition, medical aspects of the diseases, physical activity, motivation, further psychological aspects of the disease and general aspects of living with a chronic disease.

The telephone-based health coaching will be offered to all participants who score low on the Patient Activation Measure (PAM) [19] and/or low for health literacy (BHLS) [25] before the start of the intervention. Patients will be able to choose whether to accept the offer or not. If the offer is accepted, they will be given motivational interviewing by specially trained staff to compensate for the low levels of activation and/or health literacy. In the first phone call, patients’ intentions and previous experience with regard to physical activity and healthy nutrition will be identified. Based on these intentions and previous experience, the health coaches will then be able to individually address patients’ willingness to change their physical activity and nutritional behaviour during subsequent telephone calls. During these phone calls, various behaviour change techniques [28, 29] will be applied.

The patient feedback given to participants will be personalised and formulated for easy understanding. It will be based on the feedback reports for GPs that are currently submitted to general practices on a quarterly basis. During the intervention, the Central Research Institute of Ambulatory Health Care in Germany (Zi) will prepare these reports by translating routine GP data into personalised reports for the participants (see Additional Files 2 and 3). The Zi will send these reports to the GPs, who will forward them to the patients. This will allow participants to read about their changes (blood sugar, blood pressure values, weight and low-density cholesterol). They will then be able to discuss these changes with their GP and ask for appropriate adjustments. Participants also receiving telephone-based health coaching will be addressed by the telephone coaches about the patient feedback reports and will be given explanations about these reports.

The browser-based web portal will be accessible to all members of the intervention group through personalised access tokens. Evidence-based information on physical activity, nutrition and self-motivation will be available. Patients will be able to interact with structured behaviour change modules comprising multiple behaviour change techniques. Furthermore, the web portal will offer an online forum and a video-based exercise programme. The web portal will be password-protected.

#### Criteria for discontinuing or modifying allocated interventions {11b}

Each patient will be able to stop participating in intervention activities whenever they like. The activities within the PSG-meetings will be selected in agreement with the group members and will thus be tailored to their needs.

#### Strategies to improve adherence to interventions {11c}

The intervention is designed with the aim of making participation in the different intervention activities attractive. Moreover, patients will enjoy several services that they do not get outside the intervention, e.g. the browser-based web portal, the telephone-based health coaching and the patient feedback reports. Apart from this, no further measures will be undertaken to improve adherence. Any further measure would not be in line with the aim of performing a pragmatic randomised controlled trial. Attendance at the PSG-meetings will be documented by the PSG leaders, telephone calls will be documented by the telephone coaches, use of the digital components of the intervention will be documented electronically and delivery of the patient feedback reports will be documented by the Zi.

#### Relevant concomitant care permitted or prohibited during the trial {11d}

There are no restrictions on patients’ activities outside of or in addition to the intervention.

#### Provisions for post-trial care {30}

Participants will be encouraged to continue with the PSG meetings when the study is over. Trial participation is not expected to cause any harm that might require compensation.

### Outcomes {12}

#### 12.1 Outcomes

The primary outcome is defined as the number of hospital days within the last year prior to the end of the intervention. Hospital days include outpatient (e.g. emergency room or outpatient procedures) and inpatient benefits.

The secondary outcomes are the variables required for the testing of the secondary hypotheses (see the ‘Objective {7}’ section). These variables are:

- Self-reported physical health assessed using an index based on the four physical health items of the EuroQol Questionnaire with 5 dimensions and 5 levels (EQ-5D-5L) [30]
- Self-reported physical activity assessed using a modification of the Godin Leisure Time Exercise Questionnaire (GLTEQ) [31]
- Self-reported nutritional behaviour
- Intention to follow the physical activity recommendation of the WHO [24] assessed using an item that has been self-constructed in line with usual practice when constructing items of this kind [32]
- Perceived behavioural control with regard to following the physical activity recommendation of the WHO assessed using an item that has been self-constructed in slight modification of the usual practice when constructing items of this kind [33]
- Intention to perform at least five of the nine items of health-promoting nutritional behaviour described in the questionnaire
- Perceived behavioural control with regard to performing at least five of the nine items of health-promoting nutritional behaviour described in the questionnaire
- Self-efficacy with regard to health-related self-management assessed using the 4-item short version [34] of the Perceived Medical Condition Self-Management Scale (PMCSMS) [35]
- Loneliness assessed using the 6-item short-version of the Revised University of California Los Angeles (UCLA) Loneliness Scale [36, 37]
- Depression assessed using the first two items of the Patient Health Questionnaire with 4 items (PHQ-4) [38]
- Anxiety assessed using the last two items of the PHQ-4 [38]

#### 12.2 Analysis metric

The analysis metric consists in changes from baseline.

#### 12.3 Method of aggregation

Data will be aggregated via statistical means.

#### 12.4 Time points

Hospital days will be sourced from the register data of the health insurance companies. These data are updated continuously. The collaborating health insurance companies will provide these data for evaluation after the intervention retrospectively to the point in time 1 year prior to the start of the intervention. The change scores for hospital days will be computed by subtracting the number of hospital days in the year directly before intervention start from the number of hospital days in the year directly before the end of the regular intervention, i.e. directly before month 18 after intervention start. Data for the secondary outcomes will be collected via a questionnaire (1) at enrolment (baseline survey), (2) 9 months after the start of the intervention (midterm survey), (3) 18 months after the start of the intervention (end survey) and (4) 27 months after the start of the intervention (follow-up survey). The baseline survey will be performed before the study participants have been informed about their assignment to one of the two study conditions. For the questionnaire variables, the core evaluation of the intervention will be based on the change scores between the values of the baseline survey and the values of the end survey, i.e. the survey at month 18 after intervention start.

#### 12.5 Explanations

The number of hospital days has been chosen as the primary outcome because hospital days constitute a major cost factor in the treatment of T2DM and CHD. A special outcome measure referring to possible physical harms of the intervention will not be applied because of the negligible probability of physical harms being caused by the intervention. However, each of the selected outcome measures can be used to detect negative effects of the intervention.

### Participant timeline {13}

The participant timeline is presented in Table 1.

**Table 1:**
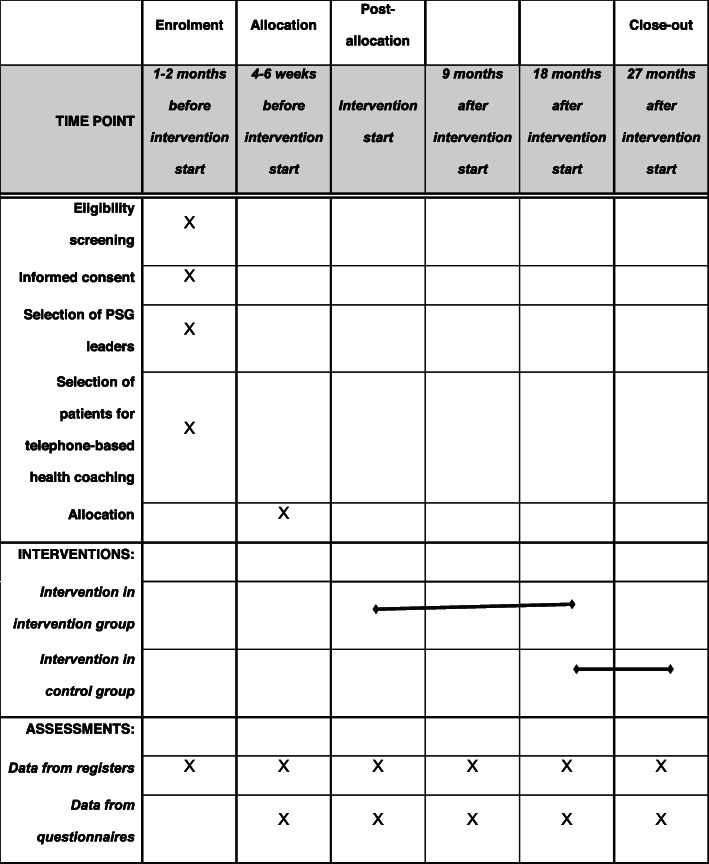
Participant timeline

### Sample size {14}

The most important analysis in the evaluation addresses the effect of the intervention on the primary outcome, i.e. the number of hospital days. As mentioned above, intervention and waiting control group will be compared with regard to the change scores of hospital days. These change scores will be computed by subtracting numbers of hospital stays 1 year before intervention start from numbers of hospital stays 1 year before the intervention end. The comparison of change scores will be performed using a *t*-test for independent samples. The sample size has been determined with regard to this test. The basic demand was that the test should be able to detect an effect of size 0.2 with a significance level of 0.05 and a power of 0.8. An effect size of 0.2 is a small effect according to Cohen [39]. However, unpublished analyses of our own with numbers of hospital days indicate that the standard deviation of change scores of hospital days will be between 5 and 10. In this case, an effect size of 0.2 would correspond to differences of one to two hospital days. Even a difference of only one day would mean a substantial difference in costs.

Further conditions presupposed for determining the sample are:

1. Two percent of the included persons could die during the intervention period or withdraw their consent for their data to be used.
2. There could be a dropout rate of 15% in the intervention group and 20% in the waiting control group. The reason for this assumption is that up to 50% of the sample might consist of persons who are not ensured at AOK RH or BARMER. For these persons, the numbers of hospital days will not be provided directly by the health insurance companies. Instead, the patients will have to request the data from their health insurance companies and then transfer them to the project team. Several patients will not do this, with patients in the waiting control group being more likely not to do this. Within this sub-sample, a dropout rate of 30% in the intervention group and 40% in the waiting control group seems realistic. This would correspond to dropout rates of 15% and 20% in the total sample.
3. The allocation ratio of intervention to control is 6:7.
4. The sample size should be divisible by 13 because of the allocation ratio.

All these considerations imply a sample size of 1001.

### Recruitment {15}

The target population will consist of patients with T2DM and/or CHD. Recruitment will continue until end of June 2021 in six areas: the cities and surroundings of Aachen, Bonn, Cologne, Düsseldorf, Essen and Bünde. All these areas are located in North Rhine-Westphalia, the federal state with the largest population in Germany. Since this study will take place in an outpatient setting, several approaches to recruiting will be applied.

The main approach will rely on the collaboration of GPs. IGKE Cologne, AMED Aachen, IFAM Bonn, SAM Cologne, ifam Düsseldorf and IFAM Essen will contact the GPs of their research and teaching practice networks as well as those in their catchment areas via postal letter and telephone calls. GPs will be included depending on their willingness to recruit patients. Staff members of the recruiting institutions will visit the included GPs. During such a visit, the participating GP and the leading practice assistant will be informed about the details of the study, how to recruit patients, how to handle the study documents and other pertinent details. They will be asked to retrieve lists of eligible patients to facilitate recruitment during office hours. They will also be asked to encourage the participation of other GPs. The GPs will approach patients who fulfil the eligibility criteria (see Eligibility criteria {10}) for participation and obtain consent from those who agree to participate. From the patients recruited, the GPs will identify suitable patients willing to volunteer as PSG leaders. Patients will be recruited successively over a period of 6 months until end of June 2021. The GPs will receive 100 € per included patient. Moreover, they will be kept completely unburdened with study-related documentation.

In addition to the main approach, AOK RH and BARMER will send personalised letters to eligible patients and already recruited patients will be asked to encourage other patients to participate. In addition, already existing self-help groups will be contacted. All measures for recruiting patients will be accompanied by announcement of the P-SUP on the Internet, in local journals that feature health-related topics, and on local radio and local television. Patients approached via these routes will be asked to contact the P-SUP study coordinator. Members of the project team will then, together with the patient, contact the patient’s GP and try to get the GP to participate.

## Assignment of interventions: allocation

### Sequence generation {16a}

The randomisation will be stratified with regard to the six areas for which the participants have been recruited and with regard to the status of the participants, i.e. PSG leader versus common PSG member. Accordingly, there will be 12 different strata combinations. For each of these combinations, a specific allocation sequence will be applied. The first author of the protocol, who is also the chief evaluator of the project, will generate these sequences. The sequences will contain blocks to ensure that the actual distribution of intervention and waiting control patients in each strata combination is always close to the envisaged ratio of 6 to 7. Moreover, to ensure that assignments to the two study conditions cannot be predicted based on the preceding assignments, the assignments will be performed weekly and those parts of the allocation sequences applied for the respective week will be always brought into a new random order.

### Concealment mechanism {16b}

The allocation sequences will only be accessible to the person who has produced them. This person will assign the study conditions to the identification numbers of the newly recruited participants without having any information beyond these numbers and beyond information regarding the corresponding strata combinations. The assignment of a study condition to a specific participant will not be communicated to participants or to persons involved in their recruitment before the participant has returned the questionnaire of the baseline survey. Consequently, there will be a delay of at least 2 weeks between recruitment of a patient and the time when the assignment of that patient to a study condition is made public.

### Implementation {16c}

The GPs will obtain the consent of the participants and, at the same time, enrol the participants. Staff members of AMED Aachen, IFAM Bonn, SAM Cologne, ifam Düsseldorf and IFAM Essen will collect the signed consent form and the corresponding address data in the practices. The signed consent form and the corresponding address data will be transmitted to the IGKE Cologne. The IGKE Cologne will assign identification numbers to the address data and send the identification numbers to TRAc Bamberg. TRAc Bamberg will assign study conditions to the identification numbers and send the resulting assignments to the IGKE Cologne. The IGKE Cologne will inform the participants of their assigned study condition.

## Assignment of interventions: blinding

### Who will be blinded {17a}

Nobody will be blinded. This also applies to those persons who perform the central analyses for evaluation, because a reasonable blinding for these persons would not be feasible. For the primary outcome variable, this will be impossible because there will be more data sets for the waiting control group than for the intervention group. For this reason, the data analysts could identify the groups based on the numbers of data sets in both groups. Moreover, in addition to the intention-to-treat analysis, the data analysts will also perform a per-protocol analysis. For this analysis, the data sets of those members of the intervention group who have actually not participated in the intervention would have to be removed, whereas there would be no change for the waiting control group. For this reason, even if somebody other than the data analysts would prepare the data for the intention-to-treat and the per-protocol analysis, the data analysts could identify the study conditions by comparing the data sets for intention-to-treat and the per-protocol analysis. For the secondary outcome variables, blinding with regard to study conditions will be impossible, because the data analysts will also perform a process evaluation with data only from the intervention group and because data from the baseline survey will also be applied for this process evaluation.

### Procedure for unblinding if needed {17b}

Not applicable

## Data collection and management

### Plans for assessment and collection of outcomes {18a}

The primary outcome, i.e. number of hospital days within the last year prior to the end and the beginning of the intervention, will be sourced from the registers of the collaborating health insurance companies. The information provided in these registers can be assumed to be reliable and valid. The secondary outcomes will be collected using questionnaires sent via postal letter. Most of the secondary outcomes will be assessed using questionnaire modules that are widely accepted in science and for which empirical evidence for their reliability and validity exists. This is the case for self-efficacy with regard to health-related self-management [34, 35], for loneliness [36, 37], for depression [38], for anxiety [38], and for self-reported physical health, which will be determined using the four physical items of the EQ-5D-5L [30]. Self-reported physical activity will be assessed using a modified version of the GLTEQ. The original form of the GLTEQ has been validated [31] and the validity of the modification of the GLTEQ (see Additional File 4) will be investigated in the context of the study. Nutritional behaviour will be assessed using a list of items of behaviour compiled by those responsible for the part of the PSG-leader course that addresses nutrition. The items referring to behavioural intention and perceived behavioural control for both physical activity and nutrition have been formulated according to principles established in social psychology.

### Plans to promote participant retention and complete follow-up {18b}

Regarding collection of data for the primary outcome (i.e. number of hospital days), no measures for promoting participant retention and completing follow-up will be required for the participants ensured at AOK RH or BARMER, because collection of these data will not depend on the cooperation of these. Participants ensured at other ensurance companies will be requested to ask their ensurance companies for the required information and to send this to the project team. Participants who do not react to the first request will get a second request 2 weeks later. Those who do not react to the second request will receive a third request. For the questionnaires an analogous procedure will be applied. For members of the intervention group, attendance at each PSG-meeting, collaboration in the telephone-based health coaching and use of the browser-based web portal will be documented.

### Data management {19}

All questionnaire data will be entered twice by two different persons. Using a software tool, a third person will then check agreement between the two data sets that result from this double entry. In those cases where the entries deviate, the third person will determine the correct entry by looking into the questionnaire. In those cases where the questionnaire answers are ambiguous, two persons will reach a decision via discussion as to what should be entered. Except for the modified GLTEQ, no range checks will be necessary for any further outcome. For the GLTEQ, values that are impossible to achieve in reality will be set as missing.

### Confidentiality {27}

Confidentiality will be ensured by the collaboration of three discrete organisational units that are all located at the IGKE Cologne. These units will be (1) the Trust Centre, (2) the Data Warehouse and (3) the Multiple Pseudonym Assignment Unit (MPAU). The Trust Centre will hold the data file in which the address data are combined with identification numbers. These numbers will be referred to as personal identification numbers. The Data Warehouse will hold data files in which the data collected during the evaluation are combined with a different kind of identification number. These identification numbers will be referred to as study identification numbers. The MPAU will generate and hold the list in which the personal identification numbers are assigned to the study identification numbers. All three organisational units are isolated from each other both digitally and in terms of personnel.

To obtain the register data from the collaborating health insurance companies (i.e. AOK RH and BARMER), the Trust Centre will send the data file containing the address data and the personal identification numbers to these companies. In addition, the MPAU will send them the list with the assignment of the personal identification number to the study identification number. AOK RH and BARMER will identify the records belonging to the address data, assign them to the study identification numbers and send the records with the study identification numbers to the Data Warehouse. The Data Warehouse, in turn, will send these data to the organisational units responsible for analysing the data.

To obtain the survey data, the Trust Centre will prepare the envelopes that contain the questionnaires with the personal identification numbers on the envelope and deliver these envelopes unsealed to the MPAU. The MPAU will paste the study identification numbers that correspond to the respective personal identification numbers onto the questionnaires, put the questionnaires back into their envelopes, seal the envelopes and deliver the sealed envelopes to the Trust Centre. The Trust Centre will then paste the addresses belonging to the personal identification numbers over the personal identification numbers on the envelopes and sends these envelopes to the study participants. The completed questionnaires with the study identification number will be returned to TRAc Bamberg, which will be responsible for data entry. TRAc Bamberg will deliver the files of questionnaire data with the study identification numbers to the Data Warehouse and solely the study identification numbers of the returned questionnaires to the MPAU. The MPAU will identify the corresponding personal identification numbers and transmit these numbers to the Trust Centre. The Data Warehouse will then transfer the questionnaire data with the study identification numbers to the organisational units responsible for analysing the data.

None of the members of the MPAU will have access to the data held by the Trust Centre or the Data Warehouse and vice versa. As a result, no staff member of these units will be able to assign the address data to the questionnaire data or to the records of the statutory health insurance companies. Moreover, none of the people analysing the data will have access to the list in which the address data are assigned to personal identification numbers. They will therefore not be able to assign the data to specific participants.

The data flow for health literacy and patient activation will be handled differently, because these data will be used to decide whether the participants are to receive telephone-based health coaching or not. The values for both variables will be transmitted, together with participants’ study identification numbers, to the MPAU. The MPAU will replace the study identification numbers with the corresponding personal identification numbers and transmit the resulting file to the Trust Centre. The Trust Centre will then replace the personal identification numbers with the identifying personal information and transmit the resulting data to those responsible for contacting study participants to offer them the telephone-based health coaching service. The participants will be informed in the survey that these data will be treated in this way.

### Plans for collection, laboratory evaluation and storage of biological specimens for genetic or molecular analysis in this trial/future use {33}

Biological specimens will not be collected in this study.

## Statistical methods

### Statistical methods for primary and secondary outcomes {20a}

To test the effect of the intervention on the primary outcome (i.e. on number of hospital days), the number of hospital days directly before the start of the intervention and the number of hospital days directly before the end of the intervention will be determined. The differences between these numbers will be computed, and the intervention and control groups will be compared with regard to these differences using a *t*-test for independent samples.

As outlined above (see the ‘Objectives {7}’ section), the effect of the intervention on the number of hospital days is assumed to be mediated by several different variables. Each step of the mediation will take some time. For this reason, there may well be no effect on the primary outcome although effects on the preceding variables are already identifiable. Such a pattern of results would also indicate an effect of the intervention. Consequently, if there is no statistically significant effect for the primary outcome, the pattern for the secondary outcomes will be applied in order to decide the effectiveness of the intervention. For this purpose, all secondary variables will be z-transformed and poled so that higher numbers mean better outcomes in all variables. All variables transformed in this way will be subjected to a two-factorial analysis of variance with the variables as a within-subjects factor and the study condition as a between-subjects factor. The intervention will be judged as effective if there is a statistically significant effect in favour of the intervention group. To judge whether the secondary outcomes actually determine the primary outcome, the relationships between secondary outcomes and primary outcome will be analysed using a cross-lagged panel approach with data from baseline and end surveys as predictors and the number of hospital days for the corresponding time intervals as criteria.

### Interim analyses {21b}

Interim analyses will be performed with the data of the midterm survey using the same statistics as in the final analyses. All members of the project will have access to these results and will participate in making the final decision to terminate the intervention.

### Methods for additional analyses (e.g. subgroup analyses) {20b}

A subgroup analysis will be performed with three different groups: (1) the PSG leaders, (2) the common PSG members not receiving telephone-based health coaching and (3) the common PSG members receiving telephone-based health coaching. The data of these subgroups will be analysed in the same way as the data for the total sample. Furthermore, to test whether there are any statistically significant differences between the results for the three groups, regression models will also be computed that refer to all participants but additionally contain terms for the interaction between study condition and subgroup. In the sense of a process evaluation, further analyses will be performed with variables that are only assessed in the intervention group and that are not listed within this protocol. Among other things, these analyses refer to the acceptance of the different intervention components and to the group dynamics within the PSGs.

### Methods in analysis to handle protocol non-adherence and any statistical methods to handle missing data {20c}

In general, participants assigned to the intervention group will be free to avail themselves of the different intervention components or not. The extent to which they do so will be documented, and the relationship of the extent of such use to changes in primary and secondary outcomes will be analysed.

Missing data are only expected in the case of questionnaire data, i.e. for the secondary outcome variables. For the secondary outcome variables that are based on sum scores, missing values will be imputed by the means of those items belonging to the respective variable for which the participant has provided data. A participant who has no baseline value for a specific secondary outcome variable will be excluded from the analyses that refer to that particular variable. However, this participant will be included in all analyses that refer to the variables for which this participant has a value. An intention to treat analysis will be performed for all participants who have a baseline value. In this intention to treat analysis, missing end values will be imputed by taking into consideration the natural development that can be expected for the respective variable for the duration of the intervention. This natural development will be estimated by computing the differences between end and baseline values of the completers in the waiting control group. Accordingly, a missing end value for a particular variable will be estimated by adding the corresponding difference estimated from completers in the waiting control group to the baseline value. Should the resulting value exceed the measurement range of the respective variable, the missing value will be imputed by the range boundary that has been exceeded.

### Plans to give access to the full protocol, participant-level data and statistical code {31c}

There are no plans of this kind.

## Oversight and monitoring

### Composition of the coordinating centre and trial steering committee {5d}

The IGKE Cologne, as the consortium leader of the project, constitutes the coordinating centre. It will also constitute the core of the steering committee, which is segmented into three different units with the IGKE Cologne participating in all of them. The first unit will mainly be concerned with the recruitment of the study participants. This unit will consist of the IGKE Cologne and the five partners responsible for the recruitment, i.e. AMED Aachen, IFAM Bonn, SAM Cologne, ifam Düsseldorf, IFAM Essen and KPPP Cologne. The second unit will mainly be concerned with the development of the intervention. This unit will consist of representatives of the IGKE Cologne, the two cooperating units of the DSHS, AMED Aachen, IFAM Bonn, SAM Cologne, ifam Düsseldorf and IFAM Essen. The third unit will mainly be concerned with the evaluation including endpoint adjudication and data management. This unit will consist of the IGKE Cologne and TRAc Bamberg.

### Composition of the data monitoring committee, its role and reporting structure {21a}

The data monitoring committee (DMC) will consist of members of the IGKE Cologne who do not work in the P-SUP project. The committee will comprise a physician, a health scientist and a health economist, all with experience in conducting studies in health services research. They will be able to identify possible safety problems. Accordingly, they will be allowed to submit an improvement of the study design and implementation. The DMC will meet regularly with the Trust Centre to monitor the conduct and safety of the study.

### Adverse event reporting and harms {22}

There will be no explicit reporting of ‘adverse events’ or ‘harms’ in the classical sense. Due to the nature of the study, no such events are expected to be caused by the intervention. Nevertheless, the PSG leaders will be asked to keep minutes of the PSG meetings. In these minutes, the PSG leaders will be required to document possible psychological and somatic abnormalities. In the event of medical emergencies, the PSG leaders will be required to alert the rescue service. Minutes will be taken of each telephone-based health coaching session. Here, abnormalities will also be recorded. In the event of psychological abnormalities, specialists within the project will directly initiate a crisis intervention. In the event of somatic abnormalities, the participant will be immediately referred to a GP or, in an emergency, to the emergency medical service.

### Frequency and plans for auditing trial conduct {23}

Trial conduct will be audited every 3 months by the scientific advisory board, which will consist of all persons working in P-SUP. In addition to this, the G-BA (i.e. the sponsor of the study) and the DMC will have the right to carry out quality assurance audits whenever they wish. The statutory supervisory authorities will have the right, under maintenance of confidentiality, to inspect the documents and reports of the study or the processes of the Trust Centre at any time.

### Plans for communicating important protocol amendments to relevant parties (e.g. trial participants, ethical committees) {25}

Necessary changes to the study design will be coordinated with the partners and sponsors involved. A corresponding amendment will be submitted to the ethics committee. After a positive decision, the amendment will be included in the study registry. Patients will also be informed if they are directly affected, i.e. if, for example, there are changes in the form of the intervention or in the use of patient data or if there are other deviations from the consent form.

## Dissemination plans {31a}

The results of the midterm analyses as well as the results of the final analyses will be submitted for publication in peer-reviewed international scientific journals.

## Discussion

The original plan for the study was developed before the advent of the COVID-19 pandemic. The pandemic rendered several of the original plans for the project obsolete. In response, several components of the intervention needed to be and actually were modified to fit the requirements defined by the pandemic. Nevertheless, not all problems arising from the pandemic could be solved perfectly. This applies especially to the recruitment of the participants. Recruitment of GPs and recruitment of patients by the GPs was impeded due to the enormously increased workload of the GPs resulting from the pandemic. In addition, a number of eligible patients became reluctant to participate because of the pandemic and because they belong to the high risk group. For these reasons, it is possible that the originally envisaged sample size of 1001 may not be achieved.

## Trial status

The study protocol (version 1.2, 03 February 2020) has been approved by the research ethics committee of University Hospital Cologne. The trial was registered in the Deutsches Register Klinischer Studien (German Clinical Trials Registry) (URL: https://www.drks.de) at the beginning of 2020 under the number DRKS 00020592 and will be conducted following the principles of the Declaration of Helsinki. The first patient was recruited in December 2020. Recruitment will presumably run until the end of June 2021.

## Supplementary Information

**Additional file 1.** Original version of informed consent.

**Additional file 2. **Example of patient feedback - original version.

**Additional file 3. **Example of patient feedback - English translation.

**Additional file 4.** Modified GLTEQ.

## Abbreviations

AAF Vienna: Abteilung für Allgemeinmedizin und Familienmedizin, Medizinischen Universität Wien (Department of General Practice and Family Medicine, Medical University Vienna)
AMED Aachen: Lehrgebiet für Allgemeinmedizin, Uniklinik RWTH Aachen (Teaching Area of General Practice, University Hospital RSTH Aachen)
AOK RH: Allgemeine Ortskrankenkasse, Rheinland/Hamburg (General Local Health Insurance, Rheinland/Hamburg)
BARMER: Barmer Health Insurance
BHLS: Brief Health Literacy Screen
CHD: Coronary heart disease
DMP: Disease management programme
DRKS: Deutsches Register Klinischer Studien (German Clinical Trials Registry)
DSHS Movement: Deutsche Sporthochschule Köln, Institut für Bewegungstherapie und bewegungsorientierte Prävention und Rehabilitation (German Sport University Cologne, Institute of Movement Therapy and Movement-oriented Prevention and Rehabilitation)
DSHS Psychology: Deutsche Sporthochschule Köln, Institut für Psychology (German Sport University Cologne, Institute of Psychology)
EQ-5D-5L: EuroQol Questionnaire with 5 dimensions and 5 levels
G-BA: Gemeinsamer Bundesausschuss (Federal Joint Committee)
GLTEQ: Godin Leisure Time Exercise Questionnaire
GP: General practitioner
IFAM Bonn: Institute of Family Medicine and General Practice, University of Bonn
ifam Düsseldorf: Institut für Allgemeinmedizin, Heinrich-Heine-Universität Düsseldorf (Institute of General Practice, Heinrich Heine University Düsseldorf)
IFAM Essen: Institut für Allgemeinmedizin, Universität Duisburg-Essen (Institute of General Practice, University Duisburg-Essen)
IGKE Cologne: Institut für Gesundheitsökonomie und Klinische Epidemiologie (Institute of Health Economics and Clinical Epidemiology), University Hospital Cologne
KPPP Cologne: Klinik und Poliklinik für Psychosomatik und Psychotherapie, Universitätsklinikum Köln (Department of Psychosomatics and Psychotherapy, University Hospital Cologne)
MPAU: Multiple Pseudonym Assignment Unit
PAM: Patient Activation Measure
P-SUP: Personalised self-management support programme
PHQ-4: Patient Health Questionnaire with 4 items
PMCSMS: Perceived Medical Condition Self-Management Scale
PSG: Peer support group
RCT: Randomised controlled trial
SAM Cologne: Schwerpunkt Allgemeinmedzin, Universitätsklinikum Köln (Teaching Unit of General Practice, University Hospital Cologne)
T2DM: Type 2 diabetes mellitus
TRAc Bamberg: Trimberg Research Academy, University of Bamberg
UCLA: University of California Los Angeles
WHO: World Health Organization
Zi: Zentralinstitut für die Kassenärztliche Versorgung in Deutschland (Central Research Institute of Ambulatory Health Care in Germany)

## References

[1] Heidemann C, Kuhnert R, Born S, Scheidt-Nave C (2017). 12-Monats-Prävalenz des bekannten Diabetes mellitus in Deutschland [12-months-prevalence of known diabetes mellitus in Germany]. J Health Monitoring.

[2] Busch MA, Kuhnert R (2017). 12-Monats-Prävalenz einer koronaren Herzkrankheit in Deutschland [12-months-prevalence of coronary heart disease in Germany]. J of Health Monitoring.

[3] World Health Organization (2016). Global Report on Diabetes. WHO Library Cataloguing-in-Publication Data.

[4] World Health Organization (2017). Cardiovascular diseases (CVDs): Key facts.

[5] GBD 2013 DALYs and HALE Collaborators (2015). Global, regional, and national disability-adjusted life years (DALYs) for 306 diseases and injuries and healthy life expectancy (HALE) for 188 countries, 1990-2013: quantifying the epidemiological transition. Lancet.

[6] United Nations. Political declaration of the high-level meeting of the general assembly on the prevention and control of non-communicable diseases. In: Sixty-sixth session of the United Nations General Assembly. New York; The United Nations; 2011.

[7] Bodenheimer T (2003). Interventions to improve chronic illness care: evaluating their effectiveness. Dis Manag..

[8] Ofman JJ, Badamgarav E, Henning JM, Knight K, Gano AD, Levan RK, Gur-Arie S, Richards MS, Hasselblad V, Weingarten SR (2004). Does disease management improve clinical and economic outcomes in patients with chronic diseases? A systematic review. Am J Med..

[9] Wagner EH, Austin BT, Von Korff M (1996). Organizing care for patients with chronic illness. Milbank Q..

[10] Coleman K, Austin BT, Brach C, Wagner EH (2009). Evidence on the Chronic Care Model in the new millennium. Health Aff (Millwood)..

[11] Stock S, Drabik A, Büscher G, Graf C, Ullrich W, Gerber A, Lauterbach KW, Lüngen M (2010). German diabetes management programs improve quality of care and curb costs. Health Aff (Millwood)..

[12] Stock S, Pitcavage JM, Simic D, Altin S, Graf C, Feng W, Graf TR (2014). Chronic care model strategies in the United States and Germany deliver patient-centered, high-quality diabetes care. Health Aff (Millwood)..

[13] Riemenschneider H, Saha S, van den Broucke S, Maindal HT, Doyle G, Levin-Zamir D, Muller I, Ganahl K, Sørensen K, Chang P, Schillinger D, Schwarz PEH, Müller G (2018). State of Diabetes Self-Management Education in the European Union Member States and Non-EU Countries: The Diabetes Literacy Project. J Diabetes Res..

[14] Lorig K (2006). Living a healthy life with chronic conditions: self-management of heart disease, arthritis, diabetes, asthma, bronchitis, emphysema & others.

[15] Best KL, Miller WC, Eng JJ, Routhier F (2016). Systematic review and meta-analysis of peer-led self-management programs for increasing physical activity. Int J Behav Med..

[16] Fisher EB, Boothroyd RI, Coufal MM, Baumann LC, Mbanya JC, Rotheram-Borus MJ, Sanguanprasit B, Tanasugarn C (2012). Peer support for self-management of diabetes improved outcomes in international settings. Health Aff (Millwood)..

[17] Gatlin TK, Serafica R, Johnson M (2017). Systematic review of peer education intervention programmes among individuals with type 2 diabetes. J Clin Nurs..

[18] Sørensen K, Van den Broucke S, Fullam J, Doyle G, Pelikan J, Slonska Z, Brand H, (HLS-EU) Consortium Health Literacy Project European (2012). Health literacy and public health: a systematic review and integration of definitions and models. BMC Public Health.

[19] Hibbard JH, Stockard J, Mahoney ER, Tusler M (2004). Development of the Patient Activation Measure (PAM): conceptualizing and measuring activation in patients and consumers. Health Serv Res..

[20] Friis K, Vind BD, Simmons RK, Maindal HT (2016). The relationship between health literacy and health behaviour in people with diabetes: A Danish Population-Based Study. Diabetes Res.

[21] Miller TA (2016). Health literacy and adherence to medical treatment in chronic and acute illness: a meta-analysis. Patient Educ Couns..

[22] Hibbard JH, Mahoney ER, Stock R, Tusler M (2007). Do increases in patient activation result in improved self-management behaviors?. Health Serv Res..

[23] Schaeffer D, Berens EM, Vogt D (2017). Health literacy in the German population. Dtsch Arztebl Int..

[24] Konerding U, Szel C. Promoting physical activity in persons with type 2 diabetes mellitus: a systematic review of systematic reviews. Patient Educ Couns. 2020 S0738-3991(20)30675-3. 10.1016/j.pec.2020.12.011. 10.1016/j.pec.2020.12.011 33358769

[25] Sand-Jecklin K, Coyle S (2014). Efficiently assessing patient health literacy: the BHLS instrument. Clin Nurs Res..

[26] World Health Organization. Global recommendations on physical activity for health. Geneve: WHO Press; 2010. 26180873

[27] Lesaffre E (2008). Superiority, equivalence, and non-inferiority trials. Bull NYU Hosp Jt Dis..

[28] Michie S, Richardson M, Johnston M, Abraham C, Francis J, Hardeman W, Eccles MP, Cane J, Wood CE (2013). (2013). The Behavior Change Technique Taxonomy (v1) of 93 hierarchically clustered techniques: building an international consensus for the reporting of behavior change interventions. Ann Behav Med.

[29] Geidl W, Semrau J, Pfeifer K (2014). Health behaviour change theories: contributions to an ICF-based behavioural exercise therapy for individuals with chronic diseases. Disabil Rehabil..

[30] Herdman M, Gudex C, Lloyd A, Janssen M, Kind P, Parkin D, Bonsel G, Badia X (2011). Development and preliminary testing of the new five-level version of EQ-5D (EQ-5D-5L). Qual Life Res..

[31] Godin G, Shephard RJ (1985). A simple method to assess exercise behavior in the community. Can J Appl Sport Sci..

[32] Ajzen I, Fishbein M (1980). Understanding attitudes and predicting social behavior Englewood Cliffs.

[33] Ajzen I, Madden TJ (1986). Prediction of goal-directed behavior: attitudes, intentions and perceived behavioral control. J Exper Soc Psychol..

[34] Wild MG, Ostini R, Harrington M, Cavanaugh KL, Wallston KA (2018). Validation of the shortened Perceived Medical Condition Self-Management Scale in patients with chronic disease. Psychol Assess..

[35] Wallston KA, Osborn CY, Wagner LJ, Hilker K (2010). The perceived medical condition self-management scale applied to persons with HIV/AIDS. J Health Psychol..

[36] Russell D, Peplau LA, Cutrona CE (1980). The revised UCLA Loneliness Scale: concurrent and discriminant validity evidence. J Pers Soc Psychol.

[37] Wongpakaran N, Wongpakaran T, Pinyopornpanish M, Simcharoen S, Suradom C, Varnado P, Kuntawong P (2020). Development and validation of a 6-item Revised UCLA Loneliness Scale (RULS-6) using Rasch analysis. Br J Health Psychol..

[38] Löwe B, Wahl I, Rose M, Spitzer C, Glaesmer H, Wingenfeld K, Schneider A, Brähler E (2010). A 4-item measure of depression and anxiety: validation and standardization of the Patient Health Questionnaire-4 (PHQ-4) in the general population. J Affect Disord.

[39] Cohen J (1988). Statistical power analysis for the behavioral sciences.

