# Supplementary material for: A pragmatic randomised controlled trial referring to a Personalised Self-management SUPport Programme (P-SUP) for persons enrolled in a disease management programme for type 2 diabetes mellitus and/or for coronary heart disease
Source: Trials. 2021 Sep 27;22:659. doi: 10.1186/s13063-021-05636-4 (PMC8475316; doi:10.1186/s13063-021-05636-4)
Supplement: Supplementary file 1 — Additional file 1. Original version of informed consent. [file 13063_2021_5636_MOESM1_ESM.pdf]

Gesamt-Studienleitung und lokal verantwortliches Zentrum

Gesamtverantwortung

Prof. Dr. Stephanie Stock
Institut für Gesundheitsökonomie und klinische Epidemiologie
Universitätsklinik Köln
Gleueler Str. 176-178
50935 Köln
Telefon: +49 221 478-30901
stephanie.stock@uk-koeln.de

Lokale Projektverantwortliche/ Projektzentren

Prof. Dr. Stephanie Stock
Institut für Gesundheitsökonomie und klinische Epidemiologie
Uniklinik Köln
Gleueler Str. 176-178
50935 Köln

Kontaktdaten:
Lisa Giesen
Tel: 0221 478 30900
Fax: 0221 478 1460340
E-Mail: lisa.giesen@uk-koeln.de

Information über die Teilnahme am Projekt

Personalisiertes Selbstmanagement Unterstützungsprogramm (P-SUP)

Sehr geehrte Dame, sehr geehrter Herr,

Sie sind durch ihren Hausarzt in einem Chroniker Programm zur besseren Versorgung Ihrer chronischen Erkrankung (Disease Management Programm DMP) eingeschrieben. Im Rahmen dieses Programms erhalten Sie eine medizinische Behandlung nach dem neuesten Stand der Forschung. Zusätzlich können Sie selbst einiges zur Ihrem Wohlergehen beitragen. Beispielsweise können Sie durch regelmäßige leichte körperliche Aktivität Ihren Blutzucker und Ihren Blutdruck senken. Um Sie dabei zu unterstützen, laden wir Sie ein, an einem neuen Projekt teilzunehmen. Dieses Projekt führen wir zusammen mit Ihrem Hausarzt und Ihrer Krankenkasse durch. Im Rahmen des Projekts treffen Sie sich einmal in der Woche mit Menschen, die eine ähnliche Erkrankung haben wie Sie selbst. Bei den wöchentlichen Treffen können Sie Erfahrungen austauschen und gemeinsame Aktivitäten unternehmen.

Ihr Hausarzt hat Sie über dieses Projekt bereits informiert. Wir möchten Ihnen die Ziele und den Ablauf kurz erklären. Lesen Sie diese Information bitte sorgfältig und gewissenhaft durch. Weitere Informationen können Sie in Ihrer Hausarztpraxis erhalten. Bitte zögern Sie nicht, alle Punkte anzusprechen, die Ihnen unklar sind. Sie werden danach ausreichend Bedenkzeit erhalten, um über Ihre Teilnahme zu entscheiden.

Ihre Teilnahme ist freiwillig. Sie können an dem Projekt teilnehmen, wenn Sie dazu schriftlich Ihre Einwilligung erklären. Sofern Sie NICHT an dem Projekt teilnehmen wollen oder später Ihre Einwilligung widerrufen, entstehen Ihnen daraus KEINE Nachteile.

Ziel der Studie

Ziel dieser Studie ist es, Sie beim Umgang mit Ihrer Erkrankung zu unterstützen und Ihre Lebensqualität zu verbessern.

Art der Studie

Im Rahmen des Projektes treffen Sie sich einmal wöchentlich mit Menschen mit der gleichen Erkrankung zum gemeinsamen Erfahrungsaustausch und leichter körperlicher Aktivität, wie z.B. aktives Spazierengehen. Um den Erfolg des Projektes messen zu können, planen wir 1664 Studienteilnehmer einzuschließen, bilden daraufhin 2 Gruppen und vergleichen diese später miteinander. Sie werden zufällig einer der beiden Gruppen zugeordnet. Die Gruppen unterscheiden sich lediglich im Zeitpunkt des Beginns. Aber beide Gruppen erhalten die gleichen untenstehenden Angebote. Beide Gruppen werden in bestimmten Abständen per Fragebogen nach Ihrem Gesundheitszustand und Ihrem Wohlbefinden befragt. Die Fragebögen dienen der Erfolgskontrolle des Projektes und der fortlaufenden Verbesserung.

In der jeweiligen Gruppe machen wir Ihnen die folgenden Angebote:

- Wöchentliche Gruppentreffen: Einmal pro Woche treffen Sie sich mit Menschen mit der gleichen oder ähnlichen Erkrankung zu leichter körperlicher Aktivität. In regelmäßigen Abständen ergänzen Vorträge- oder Gesprächsrunden diese Treffen. Sie können Themen und Referenten gemeinsam in der Gruppe festlegen.

- Online Angebot: Auf einer speziell für das Projekt erstellten Internetseite erhalten Sie aktuelle Informationen zu den Themen Bewegung und Ernährung im Alltag.

- Projektbezogenes Arzt-Patienten Gespräch: Im Rahmen Ihrer regelmäßigen Kontrolltermine in der Praxis informiert Ihr Arzt Sie anhand Ihrer persönlichen medizinischen Daten über Ihre Fortschritte bzw. die Entwicklung ihres Gesundheitszustandes.

- Telefonische Unterstützung: Wenn Sie einen besonderen Bedarf haben, bieten wir Ihnen eine telefonische Unterstützung an. Mit speziell geschultem Fachpersonal können Sie z.B. darüber sprechen, welche Art von Bewegung gut für Sie ist und wie Sie Bewegung in Ihren Alltag einbauen können.

Das Projekt wird durch den Innovationsfonds beim Gemeinsamen Bundesausschuss (G-BA) gefördert. Der G-BA ist ein Gremium, welches aus Vertretern der Krankenkassen, Ärzte und Krankenhäusern besteht.

Für das Projekt liegt ein positiver Bescheid der Ethikkommission der Medizinischen Fakultät zu Köln vor (Datum: 06.07.2020 | Zeichen: 20-1155).

Wer kann an der Studie teilnehmen?

An diesem Projekt können alle Versicherten der gesetzlichen Krankenkassen teilnehmen, die in dem Disease Management Programm für die Erkrankung Diabetes Mellitus Typ 2 und/oder Koronaren Herzkrankheit (KHK) eingeschrieben sind. Darüber hinaus sollten Sie Ihren Wohnsitz in Nordrhein haben, über ausreichende Deutschkenntnisse verfügen und physisch sowie psychisch in der Lage sein an Gruppenaktivitäten teilzunehmen.

Nicht teilnehmen können Versicherte, die nicht schriftlich eingewilligt haben oder schwere behandlungsbedürftige Folge- oder Nebenerkrankungen aufweisen.

Mögliche Risiken/Belastungen/Nebenwirkungen die mit der Teilnahme verbunden sind

Durch die Teilnahme an diesem Projekt entstehen keine zusätzlichen Risiken. Die körperliche Aktivität wird nach Rücksprache mit Ihrem Arzt und unter Anleitung von Sporttherapeuten eingeübt. Ansonsten entsprechen die Aktivitäten ihren normalen Freizeitverhalten.

Möglicher Nutzen durch die Teilnahme

Durch die Teilnahme an P-SUP kann für Sie folgender persönlicher Nutzen ergeben:

- Verbesserung Ihrer Lebensqualität durch gemeinsame körperliche Aktivität, der Teilnahme an Informationsveranstaltungen zu spezifischen krankheitsbezogenen Themen (z.B. Ernährung), Vernetzungsmöglichkeiten mit Gleichgesinnten und einer speziell für Sie entwickelten Informations-/ Lernplattform

- Stabilisierung Ihres Gesundheitszustandes, durch eine Verbesserung Ihrer medizinischen Werte, wie z.B. eine Verringerung Ihres Langzeitblutzuckers (HbA1C), Gewichtes oder Blutdrucks

- Verringerung des Risikos Begleit- und Folgeerkrankungen zu entwickeln

Studienausschluss

Unter folgenden Umständen kann es zu einer Beendigung Ihrer Teilnahme kommen:

- wenn aus medizinischer Sicht die Fortführung der Studie ein zu hohes Risiko für Sie darstellt
- wenn Sie für sich selbst oder für weitere Studienteilnehmer ein Risiko darstellen

Datenverarbeitung und Datenschutz

Im Rahmen dieses Projektes werden Daten erhoben, aufgezeichnet und an das Datencenter des Projektes und Projektverantwortliche weitergegeben. Dies schließt neben Fragebogendaten ebenfalls Leistungsdaten Ihrer Krankenkasse, sowie auch die Daten ein, die innerhalb des Chroniker Programms (DMP) erfasst werden. Zweck der Erhebung und Verarbeitung Ihrer Daten, ist die Beurteilung des Mehrwertes der im Rahmen von P-SUP angebotenen Projektbestandteilen gegenüber der Regelversorgung, die Sie im Rahmen des DMPs erhalten. Die Daten werden pseudonymisiert. Das bedeutet, dass Ihr Name nirgendwo auf den Fragebögen steht. Dies gilt auch für die Datenpakete, die an die verantwortlichen Projektmitglieder weitergegeben werden. Ihr Name wird durch eine Zufallsnummer ersetzt. Eine Zuordnung beispielsweise der von Ihnen ausgefüllten Fragebögen zu Ihrer Person wird nicht durchgeführt. Sie ist nur über eine im Studienzentrum hinterlegte Identifikationsliste möglich. Diese Identifikationsliste verbleibt im Studienzentrum und wird nicht an Ihren Arzt, die Mitarbeiter des Projektes oder Ihre Krankenkasse weitergegeben.

Die im Fragebogen erhobenen Angaben werden pseudonymisiert gespeichert und ausgewertet. Um uns ein umfassendes Bild von dem Erfolg unseres Projektes machen zu können, wollen wir auch einige bei Ihrer Krankenkasse gespeicherte Daten auswerten, wie zum Beispiel die Häufigkeit Ihrer Krankenhausaufenthalte im Projektzeitraum. Diese Daten werden selbstverständlich pseudonymisiert ausgewertet. Das heißt, dass wir bei der Auswertung Ihren Namen NICHT mit Ihren Krankenkassendaten zusammenführen dürfen.

Eine Entschlüsselung der Daten erfolgt nur im Falle der Rücknahme Ihrer Einverständniserklärung und nur zum Zwecke der Löschung Ihrer Daten.

Zum Zwecke der Prüfung der ordnungsgemäßen Durchführung der zuständigen Aufsichtsbehörde, in dem gemäß geltender gesetzlicher Bestimmungen zulässigen Maße, kann außerdem ein direkter Zugang zu Ihren medizinischen Originalaufzeichnungen gewährt werden, ohne dabei die Vertraulichkeit Ihrer Daten zu verletzen.

Die Rechtsgrundlage für die Datenverarbeitung ist Ihre freiwillige, schriftliche Einwilligung, nach Artikel 6. der DSGVO.

Die im Rahmen des Projektes erhobenen Daten werden von einem elektronischen Datensystem erfasst und statistisch ausgewertet. Nach Beendigung des Projektes werden alle Daten über einen Zeitraum von 10 Jahren in einem sicheren System gespeichert und archiviert. Im Anschluss werden Ihre Daten gelöscht.

Die Verarbeitung der erhobenen Daten erfolgt in Verantwortung der Studienleitung.

Sie haben das Recht auf Auskunft sowie Einsichtnahme über die Sie betreffenden personenbezogenen Daten, die im Rahmen des Projektes erhoben und verarbeitet werden. Dies beinhaltet die unentgeltliche Überlassung einer Kopie über die sie betreffenden personenbezogenen Daten.

Sollten Sie dabei Fehler in Ihren Daten feststellen, so haben Sie das Recht, diese berichtigen zu lassen.

Sie haben auch das Recht, eine Löschung der über Sie gespeicherten Daten zu verlangen.

Die Daten werden zu jeder Zeit vertraulich behandelt. Wissenschaftliche Veröffentlichungen von Ergebnissen erfolgen ausschließlich anonymisiert, also in einer Form, die keine Rückschlüsse auf Ihre Person zulässt.

Sind mit der Datenverarbeitung Risiken verbunden?

Bei jeder Erhebung, Speicherung, Nutzung und Übermittlung von Daten bestehen Vertraulichkeitsrisiken (z.B. die Möglichkeit, die betreffende Person zu identifizieren). Diese Risiken lassen sich nicht völlig ausschließen und steigen, je mehr Daten miteinander verknüpft werden können. Die Projektverantwortliche versichert Ihnen, alles nach dem Stand der Technik Mögliche zum Schutz Ihrer Privatsphäre zu tun und Daten nur an Stellen weiterzugeben, die ein geeignetes Datenschutzkonzept vorweisen können. Medizinische Risiken sind mit der Datenverarbeitung nicht verbunden.

Die Datenübermittlung ist nur mit Ihrer Einwilligung rechtlich zulässig. Kann ich meine Einwilligung widerrufen?

Sie können Ihre Einwilligung jederzeit ohne Angabe von Gründen schriftlich oder mündlich widerrufen, ohne dass Ihnen daraus ein Nachteil entsteht. Wenn Sie Ihre Einwilligung widerrufen, werden keine weiteren Daten mehr erhoben. Die bis zum Widerruf erfolgte Datenverarbeitung bleibt jedoch rechtmäßig.

Im Falle eines Widerrufs werden sämtliche Daten unverzüglich gelöscht oder vollständig anonymisiert.

Welche weiteren Rechte habe ich bezogen auf den Datenschutz?

Bei Anliegen zur Datenverarbeitung und zur Einhaltung der datenschutzrechtlichen Anforderungen sollten Sie sich in erster Linie an die verantwortliche Stelle (siehe Seite 1) wenden. Sie können sich auch an folgende Datenschutzbeauftragte wenden:

a) Datenschutzbeauftragter des Projektzentrums

Datenschutzbeauftragter der Universität zu Köln:
dsb@verw.uni-koeln.de
0221-470-3872

b) Datenschutzbeauftragter der Studienleitung:

Datenschutzbeauftragter der Uniklinik Köln:
datenschutz@uk-koeln.de
0221-478-88008

Sie haben außerdem ein Beschwerderecht bei jeder Aufsichtsbehörde für den Datenschutz. Eine Liste der Aufsichtsbehörden in Deutschland finden Sie unter

https://www.bfdi.bund.de/DE/Infothek/Anschriften_Links/anschriften_links-node.html

Die für die Projektleitung zuständige Datenschutzaufsichtsbehörde ist:

Helga Block
Kavalleriestraße 2-4
40213 Düsseldorf
Tel.: 0211/38424-0
poststelle@ldi.nrw.de

Aufwandsentschädigung und Kostenerstattung

Eine Aufwandsentschädigung wird Ihnen für Ihre Teilnahme an der Studie nicht gezahlt. Es entstehen Ihnen und Ihrer Krankenkasse auch keinerlei Kosten durch die Teilnahme an der Studie.

Haben Sie weitere Fragen?

Sollten Sie noch weitere Fragen zum Ablauf des Projektes, zum Datenschutz, zu Ihren Rechten, usw. haben wenden Sie sich bitte an einen der Projektverantwortlichen.

Einwilligungserklärung zur Teilnahme

- **Ich habe die Information erhalten und wurde über Wesen, Bedeutung, Tragweite und Risiken des geplanten Vorhabens informiert. Mir wurde ausreichend Gelegenheit gegeben, alle offenen Fragen zu klären. Ich habe jederzeit das Recht, weitere Informationen zur Studie zu erfragen.**
- **Ich erkläre mich freiwillig bereit, an der Studie teilzunehmen.**
- **Ich willige der direkten Kontaktaufnahme von ProjektmitarbeiterInnen in Brief- oder Telefonform ein, um projektrelevante Rückfragen zu ermöglichen.**
- **Ich habe jederzeit das Recht, ohne Angabe von Gründen von der Studie zurückzutreten, ohne, dass für mich Nachteile in der medizinischen Behandlung daraus entstehen.**

Ich habe die vollständige Teilnehmerinformation zum Projekt sowie ein unterschriebenes Exemplar dieser Einwilligungserklärung erhalten.

- ____
- Vor- und Nachname des Projektteilnehmers (in Druckbuchstaben)
- ____________________________________ ________________________________
- Ort und Datum (persönlich auszufüllen) Unterschrift des Projektteilnehmers
- **Ich habe das Aufklärungsgespräch geführt und die Einwilligung des Projektteilnehmers zur Teilnahme eingeholt.**
- ____
- Vor- und Nachname des Projektmitarbeiters (in Druckbuchstaben)
- ____________________________________ ________________________________

Ort und Datum (persönlich auszufüllen) Unterschrift des Projektmitarbeiters

Einwilligungserklärung zum Datenschutz:

Bei diesem wissenschaftlichen Projekt werden personenbezogene Daten über Sie erhoben. Die Speicherung, Weitergabe und Auswertung dieser Daten erfolgt gemäß gesetzlichen Bestimmungen und setzt vor Teilnahme an dem Projekt die folgende freiwillige Einwilligung voraus:

1. Ich erkläre mich damit einverstanden, dass im Rahmen dieses Projektes erhobene Daten in pseudonymisierter Form aufgezeichnet und pseudonymisiert (ohne Namensnennung) weitergegeben werden dürfen. Dies schließt auch die Daten ein, die innerhalb des Chroniker Programms (Disease Management Programme = DMP) erfasst werden. Die Daten werden weitergegeben an:

- **den Projektverantwortlichen**
- **das Datencenter des Projektes**

2. Ich erkläre mich damit einverstanden, dass meine Daten nach Beendigung oder Abbruch des Projektes bis zu *10 Jahre nach Projektende* aufbewahrt werden. Danach werden meine personenbezogenen Daten gelöscht oder anonymisiert.

3. Ich erkläre mich damit einverstanden, dass zum Zwecke der Prüfung der ordnungsgemäßen Durchführung der zuständigen Aufsichtsbehörde, in dem gemäß geltender gesetzlicher Bestimmungen zulässigen Maße direkter Zugang zu Ihren medizinischen Originalaufzeichnungen gewährt wird, ohne dabei die Vertraulichkeit meiner Daten zu verletzen.

4. Ich bin darüber aufgeklärt worden, dass ich jederzeit Auskunft und Einsicht in die mich betreffenden personenbezogenen Daten erhalten kann.

5. Ich bin darüber aufgeklärt worden, dass ich jederzeit die Teilnahme an der Studie beenden kann. In diesem Falle werden bereits erhobene Daten gelöscht oder vollständig anonymisiert.

6. Ich habe die Informationen zum Datenschutz zur Kenntnis genommen und willige in die Datenverarbeitung ein.

Ich habe die vollständige Teilnehmerinformation zum Datenschutz sowie ein unterschriebenes Exemplar dieser Einwilligungserklärung erhalten.

____

Vor- und Nachname des Projektteilnehmers (in Druckbuchstaben)

____________________________ ____________________________

Ort und Datum (persönlich auszufüllen) Unterschrift des Projektteilnehmers

Ich habe das Aufklärungsgespräch geführt und die Einwilligung des Projektteilnehmers zum Datenschutz eingeholt.

____

Vor- und Nachname des Projektmitarbeiters (in Druckbuchstaben)

___________________________________ ________________________________

Ort und Datum (persönlich auszufüllen) Unterschrift des Projektmitarbeiters

Kontaktauskunft:

Die Selbstauskunft verbleibt bei der Projektkoordination und wird nicht an Dritte weitergegeben. Sie dient der direkten Kontaktaufnahme von ProjektmitarbeiterInnen in Brief- bzw. Telefon-Form, um ggf. projektrelevante Rückfragen stellen zu können.

Krankenkassenzugehörigkeit: ____**(Kürzel)**

DMP: ☐ Diabetes Mellitus Typ 2 ☐ Koronare Herzkrankheit

DMP-Fallnummer zur Erstellung personalisierter Feedbackberichte Ihrer medizinischen Werte**:** ___ ___ ___ ___ ___ ___ ___ (fragen Sie Ihren Hausarzt)

____
Vor- und Nachname des Projektteilnehmers (in Druckbuchstaben)

____
Straße und Hausnummer (in Druckbuchstaben)

____
Postleitzahl und Stadt (in Druckbuchstaben)

____
Telefonnummer (Festnetz und/oder Mobiltelefon)

____
E-Mail-Adresse

____
Geburtsdatum (TT.MM.JJJJ)

Ich bin damit einverstanden, dass meine Daten zur Selbstauskunft vertraulich an ProjektmitarbeiterInnen zum Zwecke der Projektsteuerung weitergegeben werden dürfen.

________________________ ________________________
Ort und Datum (persönlich auszufüllen) Unterschrift des Projektteilnehmers
