# Supplementary material for: A pragmatic randomised controlled trial referring to a Personalised Self-management SUPport Programme (P-SUP) for persons enrolled in a disease management programme for type 2 diabetes mellitus and/or for coronary heart disease
Source: Trials. 2021 Sep 27;22:659. doi: 10.1186/s13063-021-05636-4 (PMC8475316; doi:10.1186/s13063-021-05636-4)
Supplement: Supplementary file 2 — Additional file 2. Example of patient feedback - original version. [file 13063_2021_5636_MOESM2_ESM.pdf]

Praxis Dr. med. M. Mustermann | Gleueler Str. 176-178 • 50935 Köln

Praktisch, sportlich und persönlich.

DMP-FallNr: 17221M, M, Geb.Jahr 1948, AOK

11.03.2021

Sehr geehrter P-SUP Teilnehmer,

Nachstehend finden Sie eine Auswahl Ihrer medizinischen Werte. Dieser Bericht soll dazu dienen, Ihnen eine Übersicht über die Entwicklung Ihrer Werte im Laufe des Programms zu bieten.

Das **orange Symbol** deutet auf eine **Verschlechterung** Ihrer Werte hin. Das sollte aber kein Grund sein aufzugeben, die Zeit für Veränderung ist jetzt!

Das **gelbe Symbol** deutet darauf hin, dass **keine Veränderung** stattgefunden hat. Lassen Sie sich jedoch nicht entmutigen, denn Veränderung braucht manchmal Zeit!

Das **hellgrüne Symbol** deutet auf eine **Verbesserung** Ihrer Gesundheit hin. Ausgezeichnet!

Das **dunkelgrüne Symbol** bedeutet, dass Ihre Werte im **Normbereich** liegen. Dies ist ein toller Erfolg!

Das **hellblaue Symbol** deutet darauf hin, dass Ihre Werte nicht korrekt vorliegen. Dafür entschuldigen wir uns!

Bitte besprechen Sie Ihre Werte, die außerhalb des Normbereichs liegen, mit Ihrem Hausarzt. Weitere Informationen zu den Inhalten dieses Berichts finden Sie auf der Online Plattform.

Wir wünschen Ihnen alles Gute und hoffen, dass Sie Ihre Ziele erreichen!

Gewicht

Im Folgenden wird der Verlauf Ihres Körpergewichts (in Kilogramm) abgebildet.

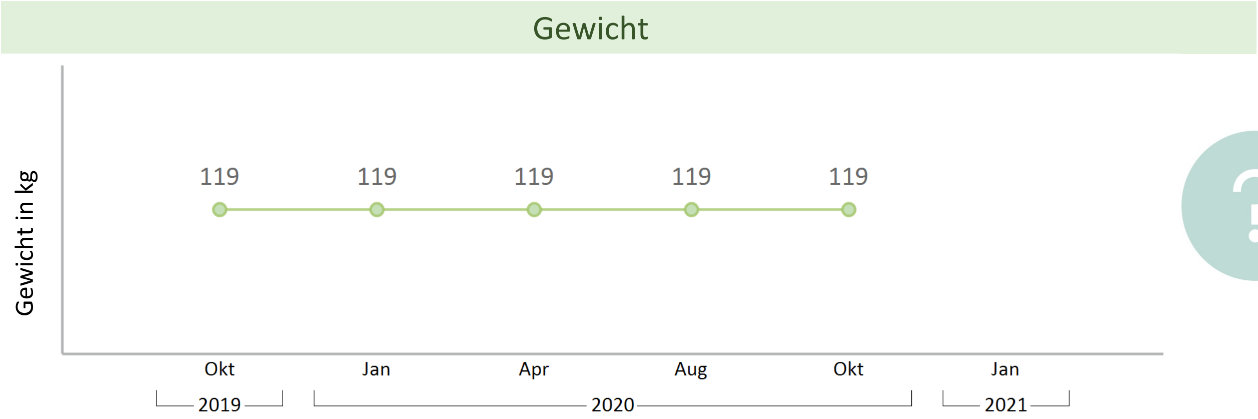

Aus dem aktuellen Quartal liegt kein gültiger Messwert vor.

Blutdruck

Ein hoher Blutdruck belastet das Herz und erhöht dessen Sauerstoffverbrauch, wodurch eine Herzkrankheit entstehen oder verschlechtert werden kann.

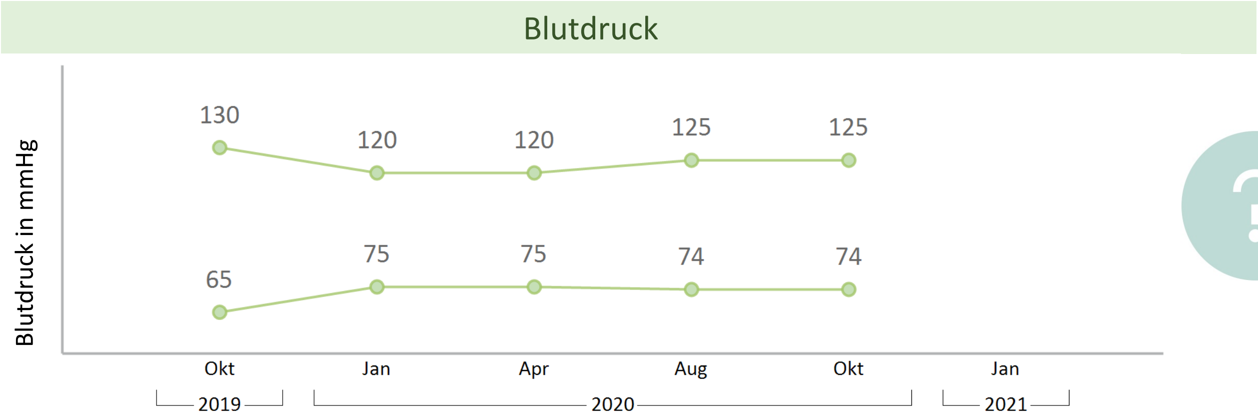

Aus dem aktuellen Quartal liegen keine Messwerte vor.

HbA1c

Dieser Blutwert gibt Auskunft über die Blutzuckerwerte der letzten 8 bis 12 Wochen.

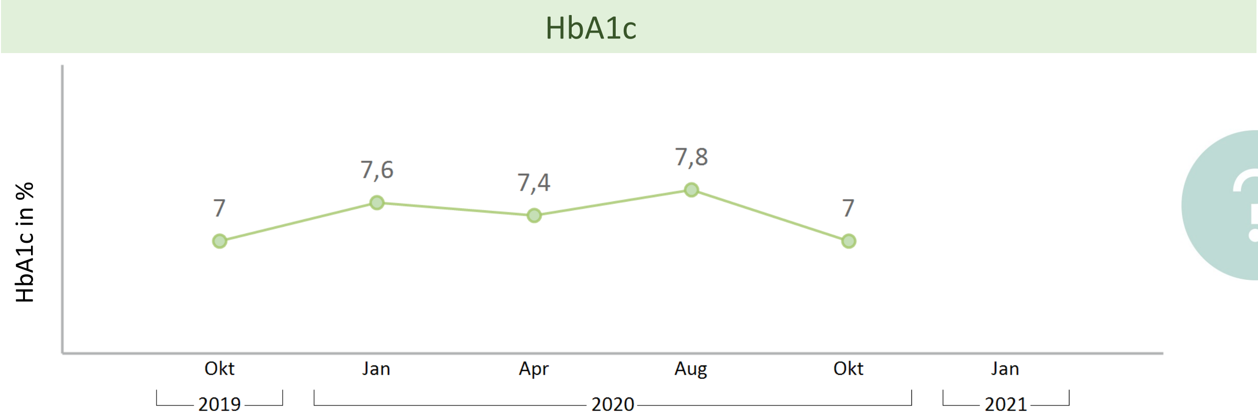

Aus dem aktuellen Quartal liegt kein Messwert vor.

LDL-Cholesterin

Hohe LDL-Cholesterinwerte schädigen die Gefäßstruktur, indem Cholesterin in die Zellen und Organe transportiert wird.

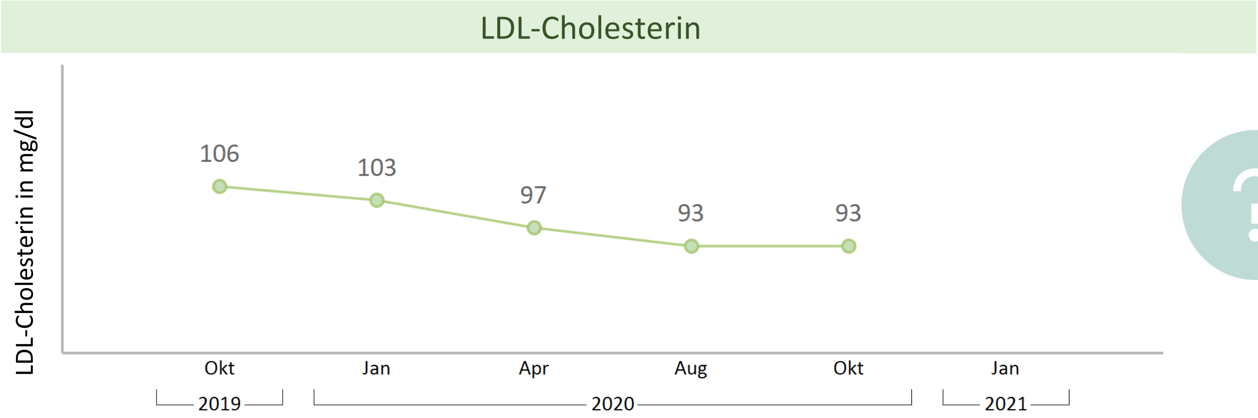

Aus dem aktuellen Zeitraum liegt kein Messwert vor.
