# Supplementary material for: A pragmatic randomised controlled trial referring to a Personalised Self-management SUPport Programme (P-SUP) for persons enrolled in a disease management programme for type 2 diabetes mellitus and/or for coronary heart disease
Source: Trials. 2021 Sep 27;22:659. doi: 10.1186/s13063-021-05636-4 (PMC8475316; doi:10.1186/s13063-021-05636-4)
Supplement: Supplementary file 3 — Additional file 3. Example of patient feedback - English translation. [file 13063_2021_5636_MOESM3_ESM.docx]

*English translation*

Logo: P-SUP – **practical, sportive and personal**

Identification line:

DMP case number, gender, year of birth, health insurance abbreviation, report number

Introduction page:

Dear P-SUP participant,

Please find a selection of your medical values below. The purpose of this report is to provide you with an overview of how your values develop over the course of the programme.

The **orange symbol** indicates a **deterioration** in your values. But that shouldn’t be a reason for you to give up. The time for change is now!

The **yellow symbol** indicates that **no change** has taken place. But don’t be discouraged. Sometimes change takes time!

The **light green symbol** indicates an **improvement** in your health. Excellent!

The **dark green symbol** means that your values are within the **normal range**. This is a real success!

The **light blue symbol** indicates that your values have not been correctly documented. We apologize for the inconvenience!

**Please talk to your family doctor about the values that are outside the normal range**. You'll find further information on the content of this report on the online platform. We wish you all the best and hope that you achieve your goals!

Page 2:

**Weight** – Your body weight (in kilograms) is shown below. No valid measured values are available from the current quarter.

**Blood Pressure** – High blood pressure puts a strain on your heart and increases its consumption of oxygen. This can cause or worsen heart disease. No valid measured values are available from the current quarter.

Page 3:

**HbA1c** – These blood values provide information on your blood sugar levels in the last 8 to 12 weeks. No valid measured values are available from the current quarter.

**LDL cholesterol** – High LDL cholesterol levels damage your vascular structure by transporting cholesterol into cells and organs. No valid measured values are available from the current period.
