# Supplementary material for: A pragmatic randomised controlled trial referring to a Personalised Self-management SUPport Programme (P-SUP) for persons enrolled in a disease management programme for type 2 diabetes mellitus and/or for coronary heart disease
Source: Trials. 2021 Sep 27;22:659. doi: 10.1186/s13063-021-05636-4 (PMC8475316; doi:10.1186/s13063-021-05636-4)
Supplement: Supplementary file 4 — Additional file 4. Modified GLTEQ. [file 13063_2021_5636_MOESM4_ESM.docx]

Appendix B:

Modified GLTEC

# German Original

| Wir würden jetzt gerne wissen, wieviel Sie sich in der letzten Zeit bewegt haben. Bitte geben Sie an, wie viele Stunden und Minuten Sie die unten beschriebenen Arten körperlicher Aktivität im letzten Monat durchschnittlich pro Woche betrieben haben.  Tragen Sie bitte hinter jeder Art körperlicher Aktivität die entsprechende Zeit ein. Wenn Sie die Art von körperlicher Aktivität gar nicht ausgeübt haben, tragen Sie bitte zweimal eine 0 ein. Eine halbe Stunde wären 0 Stunden und 30 Minuten. | | |
| --- | --- | --- |
|  | Stunden | Minuten |
| **Anstrengende körperliche Aktivität (starkes Herzklopfen)**  z.B. sehr schnelles Gehen (7 km/Std), Wandern mit Gepäck, Laufen, Sand schaufeln, schwere Last tragen, schnelles Radfahren in der Ebene (kein E-Bike; 20 km/Std), Fußball, Basketball, Hockey, Squash, Tennis, schnelles Schwimmen, Ski-Langlauf |  |  |
| **Mäßige körperliche Aktivität (leicht erhöhtes Herzklopfen)**  z.B. schnelles Gehen (5 km/Std), Boden kehren, Teppich saugen, Rasen mähen (ohne Sitzrasenmäher), Federball, Tanzen, Tischtennis, Golf |  |  |
| **Geringe körperliche Aktivität (normales Herzklopfen)**  z.B. leichtes Gehen, Betten machen, Geschirr spülen, Bügeln, Billard, Croquet, Darts, ein Musikinstrument spielen |  |  |

# English Translation

| We would now like to know how much physical activity you have had during the last time. . Please tell us how many hours and minutes of the activities described below you have performed on average per week during the last month.  Enter the corresponding time for each kind of physical activity. If you have not performed the kind of activity at all, please enter 0 twice. Half an hour would be 0 hours and 30 minutes. | | |
| --- | --- | --- |
|  | Hours | Minutes |
| **Strenuous physical activity (strong heart beat)**  e.g. very fast walking (7 km/h), hiking with baggage, running, shoveling sand, carrying a heavy load, fast cycling on level ground (no e-bike; 20 km/h), football, basketball, hockey, squash, tennis, fast swimming, cross-country skiing |  |  |
| **Moderate physical activity (slightly increased heart beat)**  e.g. fast walking (5 km/h), sweeping the floor, hoovering, mowing the lawn (no ride-on machine), badminton, dancing, table tennis, golf |  |  |
| **Light physical activity (normal heart beat)**  e.g. light walking, making the beds, doing the dishes, ironing, billiards, croquet, darts, playing a music instrument |  |  |
